# Clinical characteristics and treatment outcomes of HER2 mutation and HER2 fusion in 22 patients with advanced breast cancer

**DOI:** 10.1111/1759-7714.15130

**Published:** 2023-10-20

**Authors:** Yuxin Mu, Yanchun Meng, Yiqun Du, Xiaojun Liu, Jian Zhang

**Affiliations:** ^1^ Phase I Clinical Trial Center Fudan University Shanghai Cancer Center Shanghai China

**Keywords:** anti‐HER2 therapy, breast cancer, HER2 fusion, HER2 mutation

## Abstract

**Background:**

The clinical characteristics and efficacy of human epidermal growth factor receptor‐2 (HER‐2)‐directed agents against HER2 mutations and HER2 fusions in breast cancer are obscure due to their low frequency.

**Methods:**

We conducted a retrospective study in patients with advanced breast cancer harboring HER2 mutations and/or HER2 fusions between January 1, 2017 and January 1, 2021.

**Results:**

Among a total of 22 patients, 17 HER2 mutations were detected, including L755S, S310F, R100=, V777L, R897W, T862A, 440‐17C > G, H878Y, V842I, 73 + 9G > C, T278fs, E1069K, L755P, 226‐11C > T, 574 + 12C>T, L114V and P128L. The majority of patients had ductal carcinoma, which mostly coexisted with HER2 amplification/overexpression. The median progression‐free survival (PFS) of the 22 patients was 6.9 months (95% CI: 4.7, 9.1) in the first‐line setting. The median PFS of patients who received first‐line trastuzumab‐based regimens was significantly longer than that of patients who received a first‐line tyrosine kinase inhibitor (TKI) (10.8 months [95% CI: 2.9, 18.7] vs. 1.9 months [95% CI: 0.8, 3.0], *p* < 0.005). A total of 14 patients were treated with anti‐HER2 antibody‐drug conjugate (ADC), among whom the median treatment line of first‐time of administration of anti‐HER2 ADC was 4.5 (range, 1–10). Anti‐HER2 ADC reached an objective response rate (ORR) of 42.9%, a disease control rate (DCR) of 85.7% and a median PFS of 7.3 months (95% CI: 4.4–10.1) from the first‐time of administration.

**Conclusion:**

Our data demonstrated the clinical benefit of anti‐HER2 treatment in Chinese breast cancer patients harboring HER2 mutation and/or HER2 fusion. The value of immunotherapy and treatment selection among individual HER2 variants needs further study.

## Graphical abstract

Progression‐free survival of 22 patients in the first‐line. HER2, human epidermal growth factor receptor‐2.

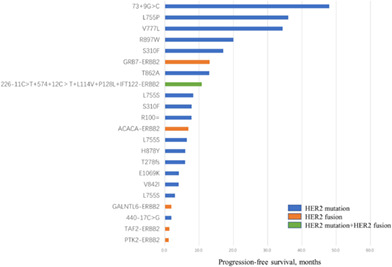

## INTRODUCTION

Human epidermal growth factor receptor‐2 (HER‐2) positive breast cancer accounts for approximately 10%–20% of all breast tumors cases.^1^, ^2^ Multiple treatment strategies targeting such HER2 amplification/overexpression have been recommended based on several large‐scale prospective clinical trials, including monoclonal antibodies, kinase inhibitors, and the antibody‐drug conjugate (ADC).^3^, ^4^, ^5^, ^6^, ^7^ The application of anti‐HER2 agents has significantly improved overall survival (OS) in the HER‐2 positive breast cancer population over the years.^8^ Other than HER2 amplification, HER2 gene mutations are also found in about 4% of multiple breast cancer types,^9^, ^10^, ^11^ exerting an oncogenic effect and indicating an additional patient group that may potentially be targeted with HER2‐directed therapies.^12^ Several studies have identified an inferior prognosis in patients with HER2 gene mutations than those without.^12^, ^13^, ^14^ HER2 gene fusions are even more rare, with few data reported. As a result of the infrequent prevalence of HER2 mutations and HER2 fusions, the clinical data of various HER2 mutations/fusions are lacking and clinical benefits of diverse treatment strategies are unclear. There is currently no established standard treatment for breast cancer patient with HER2 mutations/fusions, and exploration of an optimal treatment strategy is an area of highly unmet medical need. We conducted this retrospective study to assess the real‐world clinical impact of various therapeutic options in patients with advanced HER2 mutant/HER2 fusion breast cancer in our Cancer Center.

## METHODS

### Patient recruitment and data collection

The clinical data of patients with metastatic breast cancer who underwent circulating tumor DNA (ctDNA) analysis and in whom HER2 variants (mutations and fusions) were detected were retrospectively collected from our Cancer Center from January 1, 2017 to January 1, 2021. Eligibility criteria for registration included histologically or cytologically diagnosed metastatic breast cancer and tumor/plasma positive for HER2 mutations/fusions. Medical data of age, gender, histology, stage, hormone receptor status, HER2 expression status by immunohistochemistry (IHC), HER2 testing results by validated dual‐probed ISH assay, HER2 mutation type, HER2 fusion variant, and treatment history were retrospectively recorded. Age was recorded at initial diagnosis. Stage of disease was determined according to the American Joint Committee on Cancer (AJCC) staging system, eighth edition. To facilitate the study of HER2 mutation and the efficacy of anti‐HER2 therapy (monotherapy or combined chemotherapy) according to the different HER2 amplification and variants status, HER2 amplification‐positive was labeled as HER2+; HER2 amplification‐positive/mutation‐positive was labeled as HER2+/mut; HER2 amplification‐positive/fusion‐positive was labeled as HER2+/fus; HER2 amplification‐positive/mutation‐positive/fusion‐positive was labeled as HER2+/mut/fus; HER2 amplification‐negative/mutation‐positive was labeled as HER2−/mut; HER2 amplification‐negative/fusion‐positive was labeled as HER2−/fus. The study was conducted in accordance with the Declaration of Helsinki and the principles of Good Clinical Practice, and was approved by the Ethics Committee at Fudan University Shanghai Cancer Center.

### Assessments

The primary objective of our study was to evaluate the efficacy of chemotherapy and anti‐HER2 therapy in patients with HER2‐variant breast cancer. The primary endpoints were disease control rate (DCR) and progression‐free survival (PFS), and secondary objectives included clinicopathological characteristics of these patients and objective response rate (ORR). Disease response was evaluated according to the Response Evaluation Criteria in Solid Tumors version 1.1 (RECIST version 1.1). DCR was defined as the percentage of patients with complete response (CR), partial response (PR), or stable disease (SD), while ORR pointed to CR and PR. PFS was defined as the time interval from the date of a systemic treatment regimen (chemotherapy, or targeted therapy) initiation until date of progressive disease (PD) or death from any causes, whichever occurred first.

### Statistical analysis

The distribution of patients' baseline characteristics and treatment patterns are described. Survival analyses were analyzed using the Kaplan–Meier method and compared by the log‐rank test. Fisher's exact test was used for comparing ORR and DCR between groups. All *p*‐values were two‐sided and a *p*‐value of less than 0.05 was considered statistically significant. All statistical analyses were carried out using SPSS 23.0 statistical software (SPSS Inc.). The data cutoff was February 28, 2023.

## RESULTS

### Patient characteristics

Between January 1, 2017 and January 1, 2021, 22 female patients with HER2‐variant advanced breast cancer met the inclusion criteria and were included in our study. The median patient age was 47.7 (range, 28–70) years, 21 of 22 (95.5%) patients had ductal breast cancer, and one of 22 (4.5%) had lobular breast cancer. Among the 22 patients, five were stage I, four were stage II, 10 were stage III and three were stage IV at diagnosis. A total of 18 patients presented initially with early‐stage disease and underwent radical surgery. The median DFS after surgery of early‐stage cancers was 17.2 months (95% CI: 11.0–23.5). The clinical characteristics of the 22 patients are listed in Table 1. Of the 22 patients, five were hormone receptor (HR)‐positive/HER2‐negative, five were HR‐positive/HER2‐positive, 12 were HR‐negative/HER2‐positive, and none was HR‐negative/HER2‐ negative.

**TABLE 1:** Baseline patient demographics and clinical characteristics.

Characteristics	Patients (*n* = 22)	
	No	%
Age, years		
Median	47.7	
Range	28–70	
Sex		
Male	0	0
Female	22	100
Stage at diagnosis		
I	5	22.7
II	4	18.2
III	10	45.5
IV	3	13.6
Histology		
Ductal breast cancer	21	95.5
Lobular breast cancer	1	4.5
Molecular subtyping		
HR+/HER2‐	5	22.7
HR+/HER2+	5	22.7
HR‐/HER2+	12	54.5
HR‐/HER2‐	0	0
HER2 variants		
Fusion	5	22.7
Missense mutation	12	54.5
Synonymous mutation	1	4.5
Intron mutation	2	9.1
Frameshift mutation	1	4.5
Multiple variants	1	4.5
Metastases		
Lymph node	12	54.5
Bone	11	50.0
Liver	10	45.5
Lung	9	40.9
Chest wall	4	18.2
Pleura	1	4.5
Brain	1	4.5
Contralateral breast	1	4.5

Abbreviations: HER2, human epidermal growth factor receptor‐2; HR, hormone receptor.

### Mutation prevalence

**FIGURE 1:**
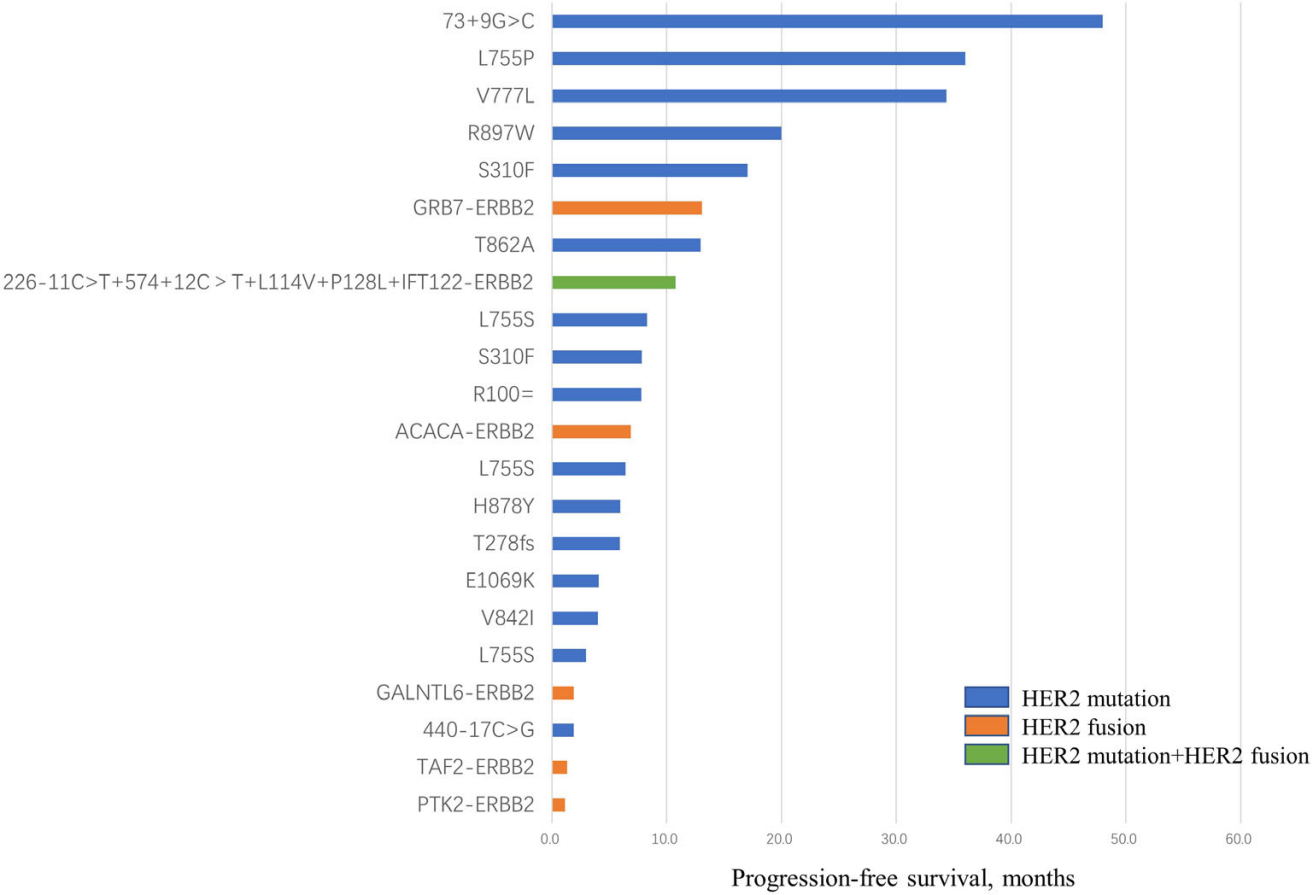
Progression‐free survival of 22 patients in the first‐line setting. HER2, human epidermal growth factor receptor‐2.

A total of 26 HER2 variants genotypes were identified (Figure 1), five patients had HER2 fusion, including TAF2‐ERBB2 (*n* = 1), GRB7‐ERBB2 (*n* = 1), PTK2‐ERBB2 (*n* = 1), GALNTL6‐ERBB2 (*n* = 1) and ACACA‐ERBB2 (*n* = 1). A total of 16 patients had HER2 mutations, including L755S (*n* = 3), S310F (*n* = 2), R100 = (*n* = 1), V777L (*n* = 1), R897W (*n* = 1), T862A (*n* = 1), 440‐17C > G (*n* = 1), H878Y (*n* = 1), V842I (*n* = 1), 73 + 9G > C (*n* = 1), T278fs (*n* = 1), E1069K (*n* = 1), L755P (*n* = 1). One patient had concomitant HER2 mutation and HER2 fusion present (226‐11C > T, 574 + 12C>T, L114V, P128L and IFT122‐ERBB2 fusion). The most common type was missense mutation (14/26, 53.8%), followed by HER2 fusions (6/26, 23.1%), intron mutation (4/26, 15.4%), synonymous mutation (1/26, 3.8%), and frameshift mutation (1/26, 3.8%). A total of 17 of 22 patients had concurrent HER2 amplification/overexpression, and five were HER2 negative. Nine cases with HER2 mutations and one case with HER2 fusion were also positive for estrogen receptor (ER) and/or progesterone receptor (PR).

### Clinical outcomes of first‐line treatment

A total of 16 HER2‐positive patients received anti‐HER2 first‐line treatments, among whom 10 received HER2‐directed antibody‐based regimen (trastuzumab ± pertuzumab in combination with chemotherapy). Five HER2‐positive patients received an anti‐HER2 tyrosine kinase inhibitor (TKI) in combination with chemotherapy, and one received HER2‐directed ADC in the first‐line setting, as they had undergone one‐year adjuvant trastuzumab‐based therapy previously and none of their DFS reached 2 years. The other one HER2‐positive patient received chemotherapy alone considering the patient's preference and access. Five HR‐positive/HER2‐negative patients received endocrine therapy as the first‐line choice.

In total, one patient had CR, 11 had PR, five had SD, and five had PD, ORR and DCR were 54.5% and 77.3%, respectively. The median PFS of 22 patients was 6.9 months (95% CI: 4.7, 9.1) in the first‐line setting. A total of 16 HER2‐directed treatments achieved a DCR of 100% for the 10 patients treated with trastuzumab‐based therapies, compared with 40% for the five patients treated with a TKI (*p* = 0.022). ORR of first‐line trastuzumab‐based therapies and TKI‐based regimens were 90.0% and 20.0%, respectively (*p* = 0.017). The median PFS of patients who received first‐line HER2‐directed antibody‐based regimens was significantly longer than that of patients who received a first‐line TKI (10.8 months [95% CI: 2.9, 18.7] vs. 1.9 months [95% CI: 0.8, 3.0], *p* < 0.005). The patient who received HER2‐directed ADC reached a PR, and a PFS of 8.3 months in the context of first‐line therapy.

### Effect of HER‐2 directed therapies

In our study, 15 patients were treated with HER2‐directed antibody trastuzumab‐based therapies (in combination with chemotherapy), and the median treatment line of first‐time of administration of trastuzumab‐based regimens was 1 (range, 1–7). The 15 patients achieved an ORR of 66.7%, a DCR of 93.3% and a median PFS of 6.9 months (95% CI: 5.2, 8.5). A total of 15 patients were treated with anti‐HER2 TKI (in combination with chemotherapy), with a median treatment line of 3 (range, 1–9) as the first‐time of receiving TKI treatment. ORR and DCR were 26.7% and 66.7%, respectively of the first‐time administration of anti‐HER2 TKI, with a median PFS of 4.1 months (95% CI: 3.5, 4.7). A total of 14 patients had treatment with anti‐HER2 ADC, among whom the median treatment line of first‐time administration of anti‐HER2 ADC was 4.5 (range, 1–10). Anti‐HER2 ADC reached an ORR of 42.9%, a DCR of 85.7% and a median PFS of 7.3 months (95% CI: 4.4, 10.1) as the first‐time of administration. Anti‐HER2 TKI in combination with trastuzumab regimen was administered in nine patients, with a median treatment line of 3 (range, 2–8) as the first‐time of receiving such combination treatment. The combination therapy demonstrated an ORR of 44.4%, a DCR of 55.6% and a median PFS of 4.7 months (95% CI: 0.0, 11.4) in the context of first‐time administration. The clinical outcomes of anti‐HER2 treatment are listed in Table 2.

**TABLE 2:** Clinical outcomes of anti‐HER2 treatment.

Treatment	Median treatment line at first‐time administration	ORR	DCR	PFS months, (95% CI)
Trastuzumab‐based regimen	1	66.7%	93.3%	6.9 (5.2, 8.5)
Anti‐HER2 TKI	3	26.7%	66.7%	4.1 (3.5, 4.7)
Anti‐HER2 ADC	4.5	42.9%	85.7%	7.3 (4.4, 10.1)
Anti‐HER2 TKI + trastuzumab	3	44.4%	55.6%	4.7 (0.0, 11.4)

Abbreviations: ADC, antibody‐drug conjugate; DCR, disease control rate; HER2, human epidermal growth factor receptor‐2; ORR, objective response rate; PFS, progression‐free survival; TKI, tyrosine kinase inhibitor.

### Clinical outcomes of HER2 mutations and HER2 fusions

Among five patients with HER2 fusions, two had PR, three had PD, with a median PFS of 1.9 months (95% CI: 0.8–3.0) in the context of first‐line therapy. The clinical outcomes of multiple HER2 fusions are listed in Table 3. A total of 16 patients harbored HER2 mutations, nine had PR, five had SD, two had PD in the first‐line setting, and responders were observed across multiple HER2 mutation types including L755S, R100=, V777L, R897W, T862A, 73 + 9G > C, T278fs, and L755P. The ORR and DCR were 56.3% and 87.5%, respectively. The median PFS of patients with HER2 mutations in the first‐line treatment was longer than those with HER2 fusions, but the difference did not achieve statistical significance (7.8 months [95% CI: 4.9, 10.7] vs. 1.9 months [95% CI: 0.8, 3.0], *p* = 0.07). One patient with concomitant HER2 mutations and HER2 fusion achieved CR on a first‐line trastuzumab‐containing regimen, PR on a second‐line lapatinib‐containing regimen, and PR on anti‐HER2 ADC treatment. A total of 12 patients were HER2+/mut in our dataset, and all were treated with trastuzumab‐based therapies. The median treatment line of the first‐time of administration of trastuzumab‐containing regimens in the HER2+/mut population was 1 (range, 1–7), with an ORR of 58.3%, a DCR of 91.7% and a median PFS of 5.9 months (95% CI: 4.1–7.8). A total of 11 of 12 HER2+/mut patients were treated with anti‐HER2 TKI, with a median treatment line of 4 (range, 1–7) as the first‐time of TKI administration. The ORR and DCR were 27.3% and 72.7%, respectively, with a median PFS of 4.1 months (95% CI: 3.2–5.0).

**TABLE 3:** Clinical outcomes of anti‐HER2 treatment upon HER2 fusions.

HER2 fusions	Trastuzumab‐based regimen		Anti‐HER2 TKI		Anti‐HER2 ADC		Anti‐HER2 TKI + trastuzumab	
	Tumor response	PFS months	Tumor response	PFS months	Tumor response	PFS months	Tumor response	PFS months
TAF2‐ERBB2	–	–	PD	1.3	SD	5.2	PD	1.6
GRB7‐ERBB2	PR	13.1	–	–	SD	8.3	–	–
PTK2‐ERBB2	–	–	PD	1.1	–	–	PD	2.1
GALNTL6‐ERBB2	–	–	–	–	–	–	–	–
ACACA‐ERBB2	PR	6.9	SD	2.8	SD	8.3	–	–

Abbreviations: ADC, antibody‐drug conjugate; HER2, human epidermal growth factor receptor‐2; PD, progressive disease; PFS, progression‐free survival; PR, partial response; SD, stable disease; TKI, tyrosine kinase inhibitor.

## DISCUSSION

HER2 is one of the most well‐studied genes in breast cancer. HER2 amplification/overexpression (HER2‐positive) is related to the occurrence and development of breast tumors, and is a prognostic indicator.^15^, ^16^ The introduction of HER2‐targeted therapies including HER2‐directed antibody, TKI and ADC has led to dramatic improvements in oncological outcomes of patients with HER2 positive tumors. In comparison, HER2 mutation is found in about 2%–4% of breast carcinomas,^10^, ^11^, ^17^, ^18^, ^19^, ^20^ and has not been well‐reported due to its low prevalence. Some studies have reported the clinical and pathological characteristics of breast cancer patients harboring HER2 mutations and/or HER2 fusions, whereas our study mainly explored the treatment pattern and clinical outcomes of various HER2 genomic subtype among these patients.

HER2 mutation has been reported to be independent of prior therapies in breast cancer^12^, ^21^ and presents with a relatively high prevalence in invasive lobular breast cancer (about 10%–15%).^22^ Approximately 70% of HER2 mutations have been reported to occur in the kinase domain, between amino acids 755 and 781 (exons 19 and 20), and about 20% were found in the extracellular domain (ECD) at either amino acid 309 or 310 (exon 8).^19^, ^21^, ^23^ Bose et al. found that HER2 R678Q, I767M, and Y835F mutations exerted no functional effects, while HER2 G309A, L755S, D769H/Y, V777L, P780_781insGSP, V842I, and R896Q mutations resulted in increased HER2 signaling and increased tumor growth in the preclinical study.^12^ Acquired HER2 mutation‐induced resistance are also very common in HER2‐positive tumors. The T798M mutation and L755S mutation have been reported to induce HER2 reactivation during treatment with lapatinib and/or trastuzumab.^24^, ^25^, ^26^ A prior study reported a higher HER2 mutation frequency in HER2‐amplified tumors than in HER2‐negative (19.5% vs. 4.8%; *p* < 0.001),^17^ and the most common V777L, L755S and D769Y mutations were all predicted as driver mutations.^17^ Our results showed that HER2 mutations equally mostly co‐existed with HER2 amplification/overexpression, while 21 of 22 (95.5%) performed in ductal carcinoma, and L755S, S310F mutations were the most common.

Furthermore, Croessmann et al. reported that 70% of HER2 mutations are identified in metastatic ER+ tumors, suggesting that the emergence of HER2 mutations may lead to endocrine therapy resistance.^27^ Several preclinical studies have demonstrated that the addition of neratinib restored the sensitivity to fulvestrant in HER2 L755S and V777L mutant breast cancer.^27^ The SUMMIT trial subsequently explored the combination of neratinib and fulvestrant in HER2 mutated, ER+ metastatic breast cancer patients. Initial results of patients treated with the combination therapy showed an ORR of 29.8%, and a median PFS of 5.4 months (95% CI: 3.7, 9.2).^28^ In our study, 10 patients were ER+, and mostly received endocrine therapy. Only one patient with HER2 V777L mutation received lapatinib combined with fulvestrant in the eighth‐line, and achieved a SD. As the outstanding clinical performances of endocrine therapy in ER+ breast cancer, the role of anti‐HER2 therapies might be mostly in more advanced treatment lines. Further studies are needed to elucidate the clinical benefits of multiple anti‐HER2 therapies in combination with endocrine therapy against HER2 mutant, ER+ breast tumors in less heavily pretreated patients.

HER2 mutations have been demonstrated to exert an oncogenic effect by constitutively activating HER2 tyrosine kinase activity or by increasing HER2 dimerization with other members of the EGFR family.^9^, ^29^ To determine whether HER2 mutation has predictive value in HER2+ breast cancer patients, we analyzed the clinical results of trastuzumab‐based therapies upon the HER2+/mut population. However, the response rates and PFS to trastuzumab‐based therapies were modest in our study. Trastuzumab resistance in cases of HER2 mutations was also detected in a prior study.^17^ In the MYPATHWAY trial, among the initial 36 patients who received trastuzumab plus pertuzumab for tumors with HER2 mutations without amplification/overexpression, no responders were found in breast cancer.^30^

Several studies reported the interaction between anticancer treatments and the immune system, and that HER2‐positive breast cancers usually contain large amounts of T cell infiltrate.^31^, ^32^, ^33^ The phase I/II trial PANACEA showed that 15% of programmed cell death 1 ligand 1 (PD‐L1)‐positive patients responded to the combination of trastuzumab with pembrolizumab, in a group of trastuzumab‐pretreated metastatic HER2‐positive patients.^34^ In the randomized phase II KATE‐2 study, the combination of T‐DM1 and atezolizumab showed a trend for an OS benefit in trastuzumab‐pretreated HER2‐positive, PD‐L1 positive population, compared with T‐DM1 monotherapy.^35^ As immunotherapy has shown activity and durable clinical benefit in patients with PD‐L1‐positive, trastuzumab‐resistant HER2‐positive breast cancer, further studies of the levels of TILs, the PD‐L1 status, and clinical benefits of immunotherapy on the HER2 mutations population are needed.

We subsequently analyzed the impact of HER2 mutations on anti‐HER2 TKI efficacy in the HER2+/mut population, considering HER2 mutations have been found to cluster in the tyrosine kinase and extra‐cellular domains of the HER2 protein and might be counterbalanced by irreversible TKI inhibitors like neratinib.^12^ Cocco et al. demonstrated that the presence of HER2 mutations can limit the sensitivity to trastuzumab and lapatinib in HER2‐amplified breast cancer patients, while remaining sensitive to neratinib monotherapy.^36^ The SUMMIT trial explored the efficacy of neratinib in HER2‐mutant tumors, and demonstrated an ORR of 32% in breast cancer patients. Responses were observed in both ER+ and ER‐, HER2‐nonamplified tumors.^37^ In our study, reversible TKIs including pyrotinib and lapatinib were mostly used in the trastuzumab‐pretreated setting, and we found that the magnitude of TKI benefits upon the HER2+/mut population was unsatisfactory compared to that of HER2 positive patients in prior studies. In phases I and II clinical trials of pyrotinib, eight of 46 patients had HER2 mutations. The median PFS of HER2‐mutant patients was shorter than that of HER2 wild‐type patients.^38^ Together, these data suggest that irreversible TKI neratinib‐based therapy may be an optional treatment choice in HER2 mutant breast cancers, regardless of copy number status of HER2 gene. Large‐scale, randomized studies are needed to validate these preliminary findings.

Trastuzumab combined with lapatinib has previously demonstrated clinical efficacy in a patient with heavily pretreated inflammatory breast cancer containing HER2 V777L and S310F mutations.^39^ Our study similarly demonstrated the clinical benefits of trastuzumab and TKI combination therapy. Considering the ability of combination therapy to overcome compensatory feedback mechanisms by more complete suppression of HER2 signaling, the potential benefit of multiple HER2‐directed therapies should also be further investigated.

A previous study demonstrated that HER2 mutations may promote the internalization of TDM‐1 regardless of the receptor levels on the cell membrane.^40^ Anti‐HER2 ADC may be more active in cancers with HER2 mutations, regardless of HER2 amplification/expression status based on this phenomenon. Overall, patients receiving anti‐HER2 ADC in our study were heavily pretreated, most of whom progressed on trastuzumab and TKI‐based regimens. The clinical outcomes of anti‐HER2 ADC in our study were fairly satisfactory, with an ORR of 42.9%, and a median PFS of 7.3 months (95% CI: 4.4–10.1) from the first‐time of administration. Since novel anti‐HER2 ADC DS‐8201a showed dramatic activity in HER2‐positive and HER2‐low breast cancers,^41^, ^42^ further studies of the new‐generation anti‐HER2 ADC in the HER2‐mutant population are warranted.

The clinical benefits of multiple HER2‐directed therapies upon the HER2‐mutant population are modest, highlighting our incomplete understanding of the biology and targetability of HER2 mutations in individual patients. Activation of downstream or parallel oncogenic pathways including HER3 mutations and the PI3K/mTOR/AKT pathway may contribute to this phenomenon, and combination therapy with downstream or parallel pathway inhibitors may lead to better outcomes.^43^, ^44^, ^45^, ^46^, ^47^

Several HER2 gene fusions were also detected in our study. The TCGA PanCancer Atlas study reported a detection rate of HER2 fusions of 1.4% in breast tumors, 1.7% in esophageal cancers, and 1.4% in cervical cancers.^40^ Given the low prevalence, the characteristics and optimal therapeutic regimen need further investigation.

Our study was limited because of its single‐center, retrospective design. The small number of cases with HER2 mutations/fusions limited the ability to draw conclusions on treatment selection and the power of interpretation to our outcomes. Furthermore, the study lacked an independent radiological review committee to re‐evaluate treatment outcomes, and thus we used DCR and PFS as our primary objectives. A multicenter, prospective study among Chinese patients harboring HER2 mutations/fusions in a larger cohort is needed.

In conclusion, our study described the clinicopathological characteristics and clinical outcomes of HER2‐directed therapies in breast cancer patients with HER2 mutations and/or HER2 fusions. The clinical benefits of trastuzumab‐based regimens and anti‐HER2 TKI were modest, while the efficacy of anti‐HER2 ADC showed promising results. The biology and targetability of HER2 mutations/fusions in individual patients, and the factors predicting treatment response need further studies.

## AUTHOR CONTRIBUTIONS

**Yuxin Mu:** Article design, data analysis, article writing and version approval. **Yanchun Meng:** Data collection. **Yiqun Du:** Data collection. **Xiaojun Liu:** Data collection. **Jian Zhang:** Article design, article revision, data collection and version approval.

## CONFLICT OF INTEREST STATEMENT

The authors declare no conflicts of interest.
